# Ready to respond: adapting rapid response team training in Papua New Guinea during the COVID-19 pandemic

**DOI:** 10.5365/wpsar.2022.13.4.981

**Published:** 2022-12-21

**Authors:** Celeste Marsh, Sharon Salmon, Tambri Housen, James Flint, Joanne Taylor, Emmanuel Hapolo, Maria Trinidad Velasco Ortuzar, Bernnedine Sissai Smaghi, Anthony Eshofonie, Berry Ropa

**Affiliations:** a University of Newcastle, Newcastle, New South Wales, Australia.; b Global Outbreak Alert and Response Network, World Health Organization, Geneva, Switzerland.; c World Health Organization Regional Office for the Western Pacific, Manila, Philippines.; d Australian National University, Acton, Australian Capital Territory, Australia.; e Hunter New England Health, New Lambton, New South Wales, Australia.; f Surveillance and Emergency Response Unit, National Department of Health, Port Moresby, Papua New Guinea.; g World Health Organization Bangladesh Country Office, Dhaka, Bangladesh.

## Abstract

**Problem:**

Rapid response teams (RRTs) are critical for effective responses to acute public health events. While validated training packages and guidance on rolling out training for RRTs are available, they lack country-specific adaptations. Documentation is limited on RRT programming experiences in various contexts.

**Context:**

In Papua New Guinea, there remain gaps in implementing standardized, rapid mobilization of multidisciplinary RRTs at the national, provincial and district levels to investigate public health alerts.

**Action:**

The human resources needed to respond to the coronavirus disease (COVID-19) pandemic forced a review of the RRT training programme and its delivery. The training model was contextualized and adapted for implementation using a staged approach, with the initiation training phase designed to ensure RRT readiness to deploy immediately in response to COVID-19 and other public health events.

**Lessons learned:**

Selecting appropriate trainees and using a phased training approach, incorporating after-training reviews, and between-phase support from the national programme team were found to be important for programme design in Papua New Guinea. Using participatory training methods based on principles of adult learning, in which trainees draw on their own experiences, was integral to building confidence among team members in conducting outbreak investigations.

**Discussion:**

The RRT training experience in Papua New Guinea has highlighted the importance of codeveloping and delivering a context-specific training programme to meet a country’s unique needs. A staged training approach that builds on knowledge and skills over time, used together with ongoing follow-up and support in the provinces, has been critical in operationalizing ready-to-respond RRTs.

## PROBLEM

The International Health Regulations (2005) require Member States to be prepared to detect and respond to public health threats and emergencies. (*1*) Core to fulfilling this requirement are public health rapid response teams (RRTs), groups of trained professionals from different disciplines with the capacity to rapidly deploy to such events. (*2*) According to the World Health Organization’s (WHO’s) *Asia Pacific strategy for emerging diseases and public health emergencies (APSED III): advancing implementation of the International Health Regulations (2005)*, (*3*) RRTs are integral to systems designed to rapidly detect and contain public health threats. Under APSED III, the key focus areas of surveillance, risk assessment and response, and therefore public health systems overall, are strengthened through the establishment of RRTs and field epidemiology training programmes (FETPs). (*4*)

There is limited published evidence on the effectiveness of well functioning RRTs during the early investigation and containment of infectious disease outbreaks and clusters. RRTs established at the local, national and international levels for West Africa during the Ebola virus disease epidemic in 2014–2016 have been credited with containing an outbreak of *Neisseria meningitidis* in Liberia in 2017 (*5*) and another Ebola virus disease outbreak in the Democratic Republic of the Congo in 2017. (*6*, *7*)

Across the WHO Western Pacific Region – where infectious disease outbreaks, emerging infectious disease threats and environmental challenges such as unsafe water and natural disasters frequently occur (*3*) – national RRTs exist in various forms and at different stages of development. A 2015 evaluation of progress towards goals under APSED III reported that substantial gains had been made in the effectiveness of the Region’s RRTs. However, countries also ranked RRTs high on their priority lists for improvement. (*8*)

To assist countries in building a trained RRT workforce, WHO’s Health Emergencies Programme produced a validated and standardized all-hazards approach training package in 2015. (*9*) In 2021, a condensed version, aimed at those working on the coronavirus disease (COVID-19) response, was developed in conjunction with the Government of India and the US. Centers for Disease Control and Prevention. (*10*) Although these are valuable resources, it is essential that such packages, including their mode of delivery, are adapted to local contexts within the Region. To support the establishment and capability of RRTs, experiences with training and the lessons identified should be documented. We present a model of RRT training and capacity strengthening being piloted in provinces across Papua New Guinea.

## CONTEXT

In Papua New Guinea, public health threats are compounded by a population of 8.8 million that is widely spread across extremely diverse terrain, with 80–85% of people living in rural or geographically isolated settings. (*11*) In recent years, Papua New Guinea has faced many major public health events, including outbreaks of measles (2017) and cholera (2019), the re-emergence of vaccine-derived poliovirus (2018) and the current COVID-19 pandemic. These outbreaks occur in addition to a high baseline burden of endemic diseases and natural disasters that have public health impacts.

**Fig. 1:**
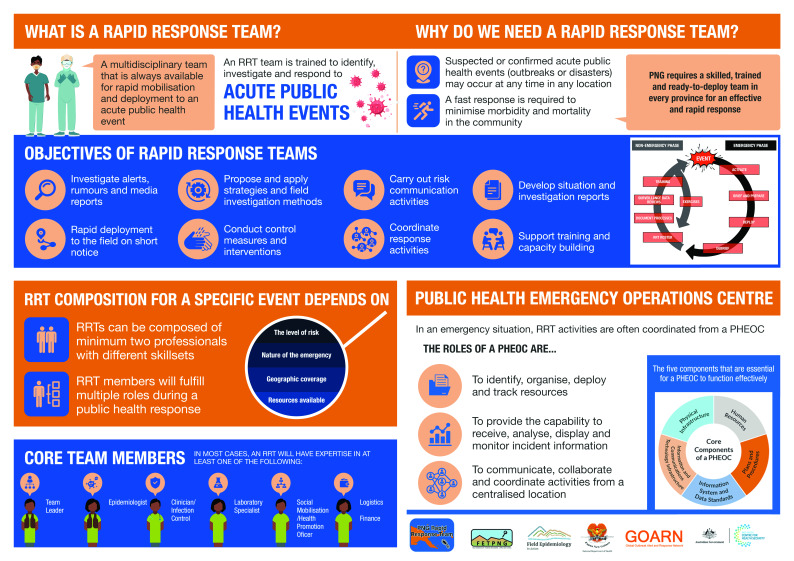
Infographic of the Rapid Response Team training process distributed to Provincial Health Authorities across Papua New Guinea, February 2022

Papua New Guinea has an established FETP that has trained 96 field epidemiologists between 2013 and June 2022. The programme has strengthened prevention, detection and response capabilities throughout the country. (*12*) FETP Papua New Guinea (FETPNG) graduates often take lead roles in outbreak responses and surveillance initiatives. However, there remain gaps at the national, provincial and district levels in the standardized, rapid mobilization of multidisciplinary teams to investigate public health alerts. In recognition of these gaps, the RRT training programme for multidisciplinary RRTs within Provincial Health Authorities was conceived and initiated by national leaders in field epidemiology. The vision was for RRTs to be operationalized through national and provincial emergency operation centres (EOCs) under the leadership of an appointed incident manager (**Fig. 1**). EOCs were first established to support the response to the polio outbreak in three provinces (the Eastern Highlands, Madang and Morobe), and they were subsequently set up in other provinces.

[**insert** Figure 1]

## ACTION

In November 2019, on request from the National Department of Health, the WHO Regional Office for the Western Pacific led an RRT training for the National Capital District Provincial Health Authority using the WHO all-hazards approach package. (*9*) With the arrival of the COVID-19 pandemic in March 2020, the need to establish functional RRTs in other provinces became urgent. An RRT programme team – composed of representatives from the National Department of Health; FETPNG; Field Epidemiology in Action, from the University of Newcastle, Australia, and Hunter New England Health, Australia; and the WHO Representative Office for Papua New Guinea – collaborated to accelerate the roll out of training across the country. This began with an after-training review with the National Capital District RRT in May 2020 that highlighted key limitations of the initial training and the need to codevelop a training package tailored to the Papua New Guinea context.

Human resources limitations in the context of the pandemic meant that it was not feasible to remove teams from ongoing response operations for 5 days of training. Therefore, the training was converted to a 2-day initiation training that covered the basic principles and structure of an RRT to assist provinces in quickly mobilizing teams to respond to both COVID-19 and other public health events. Initiation training was aimed at incident managers, disease control officers, clinicians, surveillance officers, laboratory scientists, environmental health officers, logisticians, finance administrators, risk communication specialists and health promotion officers. Graduates of FETPNG filled one or more of these roles and were the primary provincial contact points for coordinating the training. Graduates working at the National Department of Health served as trainers.

**Fig. 2:**
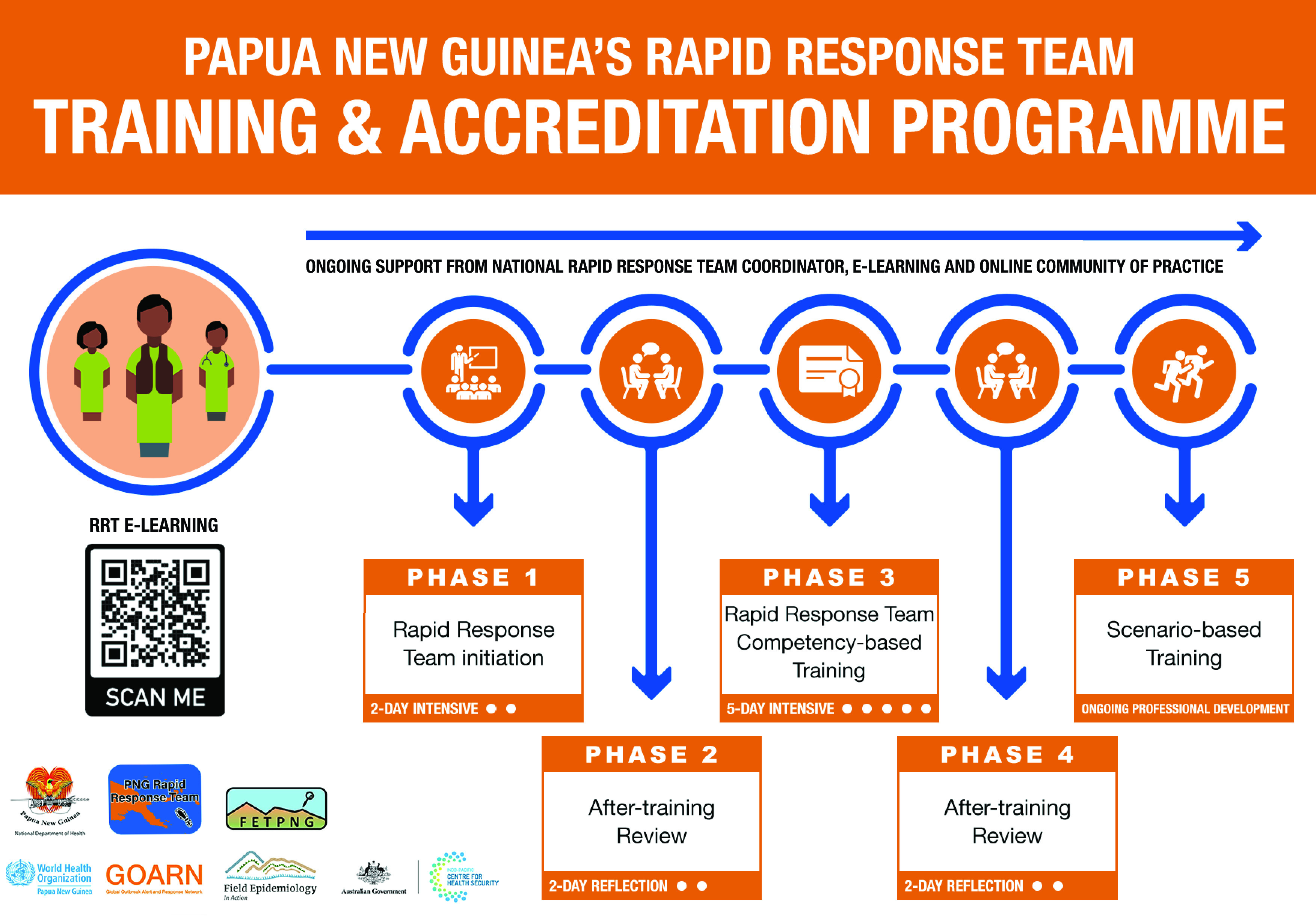
Rapid Response Team training model, Papua New Guinea, May 2022

Building on the initiation training, the RRT programme team codeveloped a phased delivery approach that provided an iterative and sequential pathway to establishing and strengthening RRTs over five phases (**Fig. 2**). Initiation training (Phase 1) facilitates the establishment of an RRT and is the first phase towards RRT accreditation. After-training reviews (Phases 2 and 4) support reflections by RRTs on their prior training and post-training implementation, and the development of action plans for improving operationalization of the teams. Competency-based training (Phase 3) focuses on extending multidisciplinary skills and knowledge. Ongoing scenario-based training (Phase 5) provides the opportunities for RRTs to keep current their early detection and response skills, continue to practice after-action reviews and refine RRT operationalization in different emergency contexts. Additionally, during Phase 5, neighbouring provincial teams come together to strengthen people-to-people links, collaboration and the regional community of practice.

[**insert** Figure 2]

In April 2021, to facilitate the development of a national RRT community of practice, the National Department of Health’s RRT coordinator set up a group channel on the encrypted messaging service WhatsApp. This platform has enabled members to seek out information and share ideas about potential clusters or outbreaks. In June 2022, the platform was used to discuss a possible multiprovincial pertussis outbreak.

RRT e-learning modules are being designed to complement the various phases of the training programme. These provide an introduction for RRT members who are unable to attend in-person trainings and act as refreshers for those who have attended. Although these e-learning modules reflect the context in Papua New Guinea, the training has been made available globally to support RRTs in similar contexts (e.g. on the Field Epidemiology in Action web site at https://www.fieldepiinaction.com and the Global Outbreak Alert and Response Network training site at https://extranet.who.int/goarn).

RRT programme activities are led and coordinated by national FETPNG faculty with the support of Field Epidemiology in Action, the WHO Global Outbreak Alert and Response Network and the WHO Representative Office for Papua New Guinea. The programme is funded by the Indo-Pacific Centre for Health Security, Department of Foreign Affairs and Trade, Australia, and WHO.

## LESSONS LEARNED

As of June 2022, RRT initiation training (Phase 1) had been conducted in 11 provinces, training 190 RRT members. The programme aims to complete initiation training in all 22 provinces by the end of 2022, with content and delivery continuing to be adapted based on ongoing monitoring and evaluation activities. Where initiation training conducted in 2020–2021 took a just-in-time training approach in response to the pandemic and included specific material related to COVID-19, training in 2022 has pivoted to focus on roles and responsibilities within RRTs.

Initiation training has been well received. Evaluations from training in the first six provinces showed most participants agreed that the RRT outbreak manual was useful (94%, 49/52), the content was relevant to their work (84%, 59/70) and the training was interactive (81%, 55/68). The most common reaction to training by participants – particularly the provincial surveillance and disease control officers, who often felt they carried the rapid response burden alone – was relief that a multidisciplinary team was being formalized and trained.

Five after-training reviews were conducted before June 2022. Factors that had reportedly worked well since initiation training included applying knowledge in the field: the steps of an outbreak investigation, pre-deployment preparation, the use of checklists and, in one province, the use of psychological first aid in a response to a landslide. Reported challenges included developing the RRT manual for the province, ensuring the most appropriate people were selected for training, assigning RRT roles and responsibilities, and accessing timely financial and logistical support for deployment. Through root cause analysis, RRTs found that progress had been hindered by factors such as team diversion to the COVID-19 response, lack of engagement by management, and human resources constraints, which led to many members straddling multiple roles. After-training reviews have identified critical challenges and best practices to help RRTs develop their own action plans.

The commitment of senior leadership to the operationalization of provincial RRTs was found to be essential to establish functional teams. Senior provincial leaders were invited to the trainings, but their presence varied across provinces. Greater effort is needed to communicate early with provincial leaders about the purpose and significance of their presence at the training.

The importance of equipping RRT trainers with skills and knowledge about teaching adults was recognized early in the roll out of initiation training. In May 2022, RRT trainers and FETPNG faculty attended a train-the-trainers workshop focused on revising training materials and methods of delivery to facilitate adult learning based on Kolb’s learning theory. (*13*) Lessons learned from this training have been applied to amend the 2-day initiation training and will be applied to the development of Phases 3–5.

In May 2022, a new position within FETPNG was created for a national RRT coordinator, and this has been of significant value to the programme. The role includes conducting ongoing post-training follow-up with provincial RRTs; coordinating training activities, monitoring and evaluation; and following up on alerts received through RRT social networks.

## Discussion

Provincial public health RRTs are a critical component of Papua New Guinea’s public health emergency response architecture and are key to strengthening health security in a context where epidemic and endemic diseases continually challenge the country’s fragile public health system. While validated training packages (*9*) and guidance on rolling out training and managing RRTs (*2*) are available, experiences in RRT programming in various contexts should be documented. The experience in Papua New Guinea has highlighted the importance of developing a training programme that meets the needs of a country and its provinces and shifts away from a tick-the-box training model towards a comprehensive, staged training approach that builds knowledge and skills over time.

The staged training approach, which includes multiple after-training reviews and ongoing scenario-based training, reflects the US. Centers for Disease Control and Prevention’s recommendation that individuals involved in RRT undergo “regular, continual training throughout their membership on the RRT.” (*14*) The planned, intermittent, scenario-based training aligns with one of the priorities set for the Western Pacific Region in APSED III that advises Member States to “conduct after-action reviews or simulation exercises that test the readiness of the national surveillance and response system as a whole to respond to outbreaks and other acute public health events.” (*3*)

The application of adult learning principles to the design and delivery of training allows RRT members to share their lived experience of the principles being taught and ensures the training is relevant to the local context in which it is delivered. In Papua New Guinea, the training programme continues to evolve based on findings from the after-training reviews, which have been essential to understanding the contextual challenges in establishing and implementing functional RRTs.

An evaluation of RRTs conducted across 21 countries between 2016 and 2018 found that they faced common challenges in establishing and managing the teams, including developing rosters for RRTs, ensuring that RRT members are trained, and that adequate standard operating procedures are lacking. (*14*) That these factors were also common challenges in the provinces of Papua New Guinea, where after-training reviews were conducted, emphasizes the importance of ensuring ongoing support from the national programme team throughout the programme cycle and beyond.

## Conclusions

Papua New Guinea has one of the world’s most rural and remote populations, so it is challenging to rapidly mobilize response teams. The development of a highly contextualized training programme built on adult learning theory and delivered in a phased approach is an important part of strengthening Papua New Guinea’s health security. Important lessons have been and will continue to be learned as the roll out of the RRT programme progresses. We hope the approach taken in Papua New Guinea and the lessons learned will be of benefit to RRT training programmes in similar contexts.
